# Supplementary material for: Dietary Patterns and Their Sociodemographic and Lifestyle Determinants in Switzerland: Results from the National Nutrition Survey menuCH
Source: Nutrients. 2018 Dec 29;11(1):62. doi: 10.3390/nu11010062 (PMC6356790; doi:10.3390/nu11010062)
Supplement: Supplementary file 1 [file nutrients-11-00062-s001.pdf]

Food categories	Food items included
Non alcoholic beverages	Sweetened soft drinks, Energy drinks, Fizzy drinks, Ice tea, Diluted syrup, Fruit juices, Drinks made with fruits juices, Smoothies, Vegetable juices, Alcohol-free beers<br>Except: non-caloric sweetened drinks
Alcoholic beverages	Beer, Wine, Wine products, Liquors, Cocktails, Spirits
Cereals & starchy	All bread products, Pasta (plain or stuffed), Rice, Spätzle, Potatoes, Sweet potatoes, Potatoe-based products (French fries, gnocchi,...), Cereal flakes and bran, Other cereal grains (Quinoa, Barley, ...), Flours, Starches, All types of dough
Red & processed meat	Fresh meat and offal of mammals (Beef, Veal, Pork, Lamb, Mutton, Horse, Goat, Rabbit), Wildmeat, Sausages, Smoked and cured meat, Meat-based spreads, Meat-based sauces
White meat	Fresh meat and offal from chicken, turkey, duck, goose, ostrich, frog
Fish	Fish, Seafood, Processed fish (Fish in crumbs, ...), Processed seafood (Surimi, ...)
Fruits & nuts	All fruits (raw, cooked, dried, in compote, in pies), Dried nuts and seeds (Almonds, Coconut, Pumpkin Seeds, ...), Olives, Avocadoes.<br>Except: Fruit juices, fruit jams, candied fruit, small amounts of fruits contained in yogurts, cakes, ice-cream or other sweets.
Vegetables	All vegetables, green leaves, sprouts, mushrooms, seaweeds, sweet corn, snow peas, fresh green beans, and onions: raw, cooked, dried, canned, in puree, pickled, in sauce (e.g. tomato sauce), on pizza and quiches, and in sandwiches. Legumes.<br>Except: avocadoes, olives, herbs, vegetable juices, or if contained in small amounts in salty snacks, bread, nor sauces.
Soups	Vegetable soups, Vegetable or meat broth, Miso soups, Mushroom soups
Added fats	Butter, Other animal fats, Vegetable oil (including oil in dressings), Margarine, Mammal's cream
Eggs	Eggs (all consumption forms), including eggs in recipes
Milk & dairy	Mammals’ milk, branded fermented milk drinks, yogurt drink and buttermilk, in liquid form, Mammals’ yogurt, branded fermented milk and kefir, fresh cheese (Quark, Cottage cheese, Ricotta, ...), Mammals’ spread cheese, processed/melted cheese, and soft cheese, Mammals’ hard cheese.
Chocolate	Chocolate chips, Chocolate tablets, Chocolate powder, Chocolate icing, Sugar, Syrup, Jams, Honey
Cakes	Cakes, Biscuits, Pies, Strudel, Muffins, Waffles, Ice cream, Sorbet, Sweet pastries, Doughnuts
Savoury snacks	Cocktail canapés, Crisps, Salty popcorn, Dried salty crackers , Crostini/bruschetta, Spring rolls, Ham croissants, Samosa and Sausage rolls.
Sauces & seasoning	Sauces rich in oil or butter (Mayonnaise, Pesto, Salad dressing, ...), Other sauces rich in cream or other fats (Cocktail sauce, Hummus, Satay sauce), Mustard, Wasabi paste.
Others	Milk and yogurt substitutes (Soya milk, ...), Meat substitutes (Quorn, Vegetarian sausages, Tofu, ...), Dietetic products rich in proteins (Meal replacements, Protein shakes, ...).

Supplementary table 1: Description of the foods and beverages included in the 17 study categories
This table is adapted from Chatelan *et al.* (Nutrients, 2017)

	Swiss traditional	Western 1 - soft drinks and meat	Western 2 - alcohol, meat and starchy	Prudent
**Participants; N**	744 (36.2%)	383 (18.6%)	444 (21.6%)	486 (23.6%)
**Sex**				
Males	38.8%	56.4%	59.2%	34.0%
Females	61.2%	43.6%	40.8%	66.0%
**Age groups[1]**				
18-29 years old	17.6%	27.4%	21.4%	14.2%
30-44 years old	26.3%	29.8%	25.0%	23.0%
45-59 years old	30.1%	29.5%	28.8%	32.9%
60-75 years old	25.9%	13.3%	24.8%	29.8%
**BMI categories[2]**				
Underweight (BMI < 18.5 kg/m^2)	2.7%	2.1%	1.6%	3.3%
Normal (18.5 ≤ BMI < 25 kg/m^2)	57.1%	50.9%	50.9%	55.3%
Overweight (25 ≤ BMI < 30 kg/m^2)	30.1%	29.2%	34.9%	28.4%
Obese (BMI ≥ 30 kg/m^2)	10.1%	17.8%	12.6%	13.0%
**Language region[3]**				
German-speaking	71.8%	66.3%	61.7%	57.4%
French-speaking	19.8%	26.4%	22.5%	31.7%
Italian-speaking	8.5%	7.3%	15.8%	10.9%
**Nationality**				
Swiss	78.4%	70.5%	72.1%	65.6%
Swiss binationals	12.6%	14.6%	12.8%	18.5%
Other	9.0%	14.9%	15.1%	15.8%
**Education, highest degree**				
Primary school or no degree	3.8%	4.7%	4.1%	5.1%
Secondary	46.5%	49.6%	50.9%	42.4%
Tertiary	49.6%	45.4%	45.0%	52.3%
**Marital status; %**				
Single	28.8%	37.9%	35.1%	24.5%
Married	60.2%	50.7%	49.8%	53.9%
Divorced	8.6%	9.4%	11.0%	15.2%
Other	2.3%	1.8%	4.1%	6.2%
**Gross household income (CHF/month)**				
< 6,000	17.3%	14.6%	18.2%	16.5%
6,000 to 13,000	41.1%	43.9%	39.2%	39.7%
> 13,000	14.5%	13.8%	13.3%	13.4%
Imputed	27.0%	27.7%	29.3%	30.5%
**Self-reported physical activity**				
Low	11.0%	15.1%	11.5%	12.3%
Moderate	22.7%	18.3%	22.5%	23.9%
High	40.9%	40.2%	39.6%	39.7%
Imputed	25.4%	26.4%	26.4%	24.1%
**Smoking**				
Never smoker	52.0%	43.3%	33.1%	44.0%
Former smoker	34.1%	25.1%	37.2%	35.6%
Current smoker	13.7%	31.1%	29.7%	20.2%
**Self-reported health**				
Very bad to medium	11.7%	12.8%	16.9%	12.6%
Good to very good	88.2%	86.7%	83.1%	87.2%
**Currently on a weight-loss diet**				
No	95.4%	95.3%	96.4%	89.9%
Yes	4.4%	4.2%	3.6%	9.9%

Supplementary table 2: Characteristics of the study participants by dietary pattern

[1] Age is the self-reported age on the day the dietary and physical activity behavior questionnaire was filled.

[2] BMI was obtained from measured height and weight. Self-reported weight or height were used when measurements were impossible. For lactating and pregnant women, self-reported weight before pregnancy was used to calculate BMI.

[3] German-speaking region includes the cantons of Aargau, Basel-Land, Basel-Stadt, Bern, Lucerne, St. Gallen, Zurich; French-speaking region: Geneva, Jura, Neuchatel, Vaud, and Italian-speaking region: Ticino.

Number of imputed values are not shown for variables with less than 0.2% of missing values (0 to 4).
Abbreviations: 24HDR, 24-hour dietary recall; CHF, Swiss Francs

A

Method	Criteria	Number of dimensions to retain	See panel
Scree plot	Break(s) in scree plot of eigenvalues over the dimensions	3, possibly 6	B
Eigenvalues	Dimensions associated with eigenvalues > 1	3	B
Guttman-Kaiser rule	Dimensions associated with an eigenvalue above the mean of all eigenvalues	15	B
Joliffe rule	Dimensions associated with an eigenvalue above 70% of the mean of all eigenvalues	26	B
Log-eigenvalue diagram	Log of eigenvalues corresponding to 'noise' should decrease linearly	6	C
Total variance	Other authors with similar approach chose dimensions associated with at least 25% of the total variance	at least 5	D
Velicer Minimum Average Partial (MAP)	Dimension associated with the lowest MAP	4	E

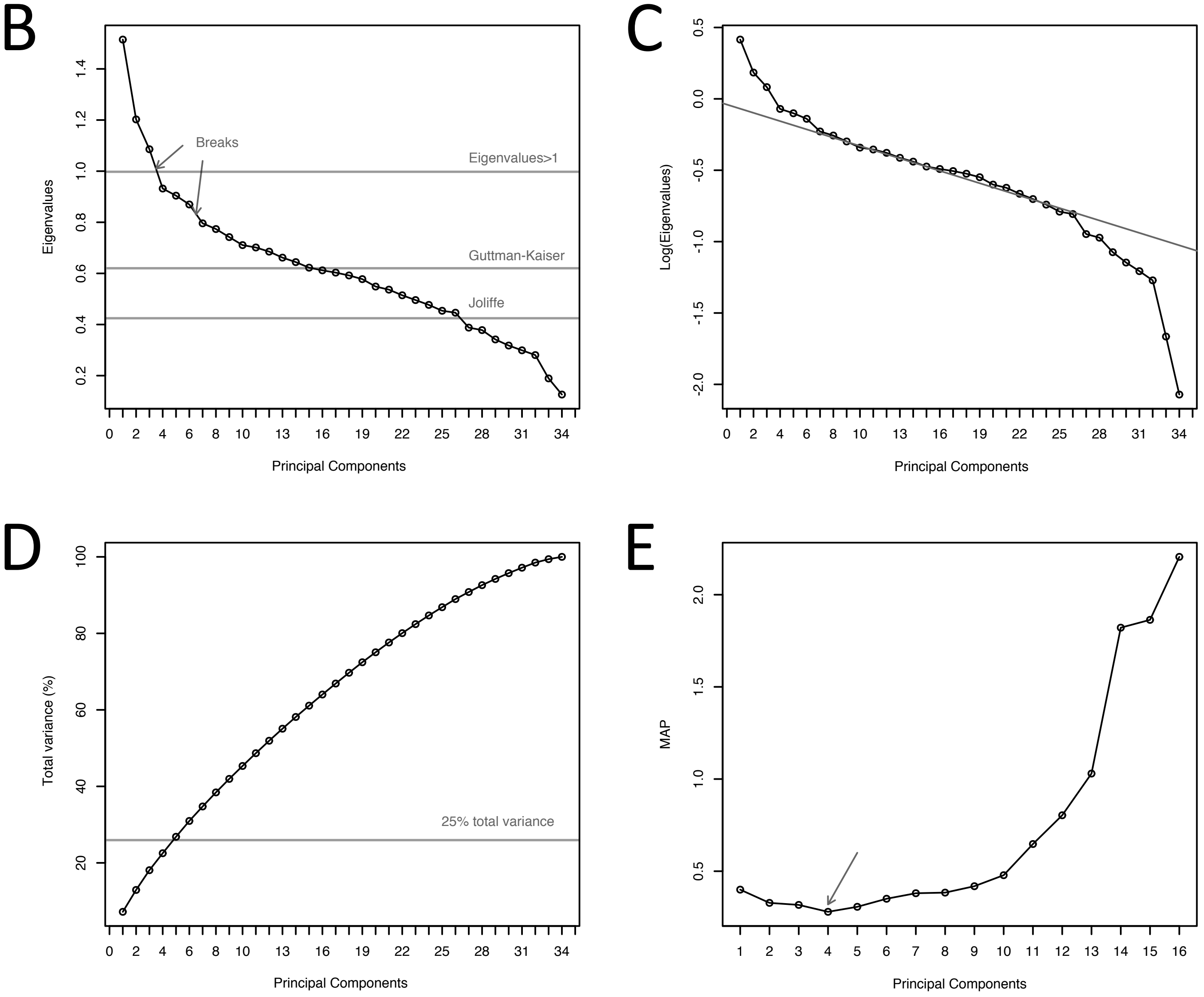

Supplementary figure 1: Synthesis of approaches used to choose the number of principal components to retain

A. Summary table of the stopping rules used. B. Tree plot, C. Log-eigenvalue diagram, D. Total variance explained and E. Velicer's Minimum Average Partial (MAP) of the multiple factorial analysis.

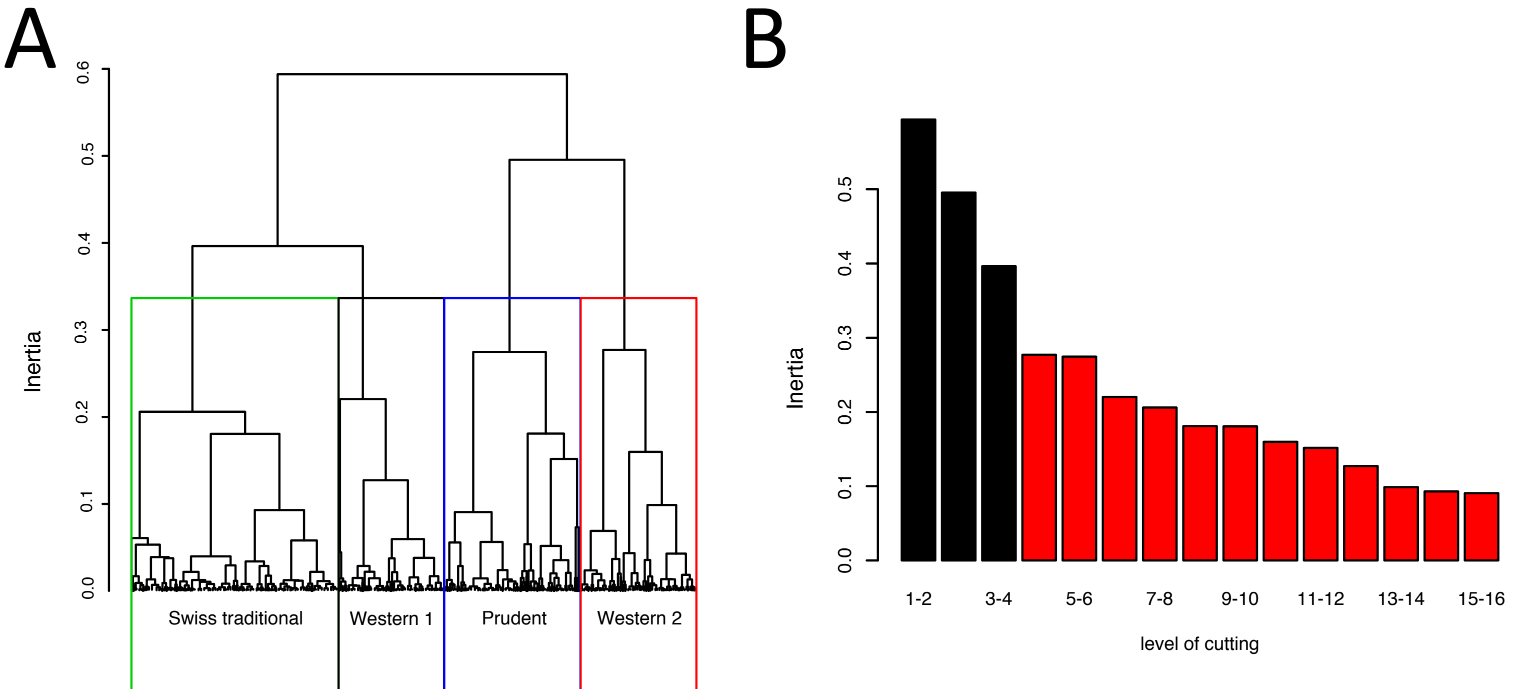

Supplementary figure 2: Changes in inter-cluster inertia indicates a robust partition with four clusters

A. Cluster dendogram obtained by hierarchical clustering on the seven first principal components of the multiple factorial analysis. B. Changes in inter-cluster inertia between partitions containing n or n+1 clusters.

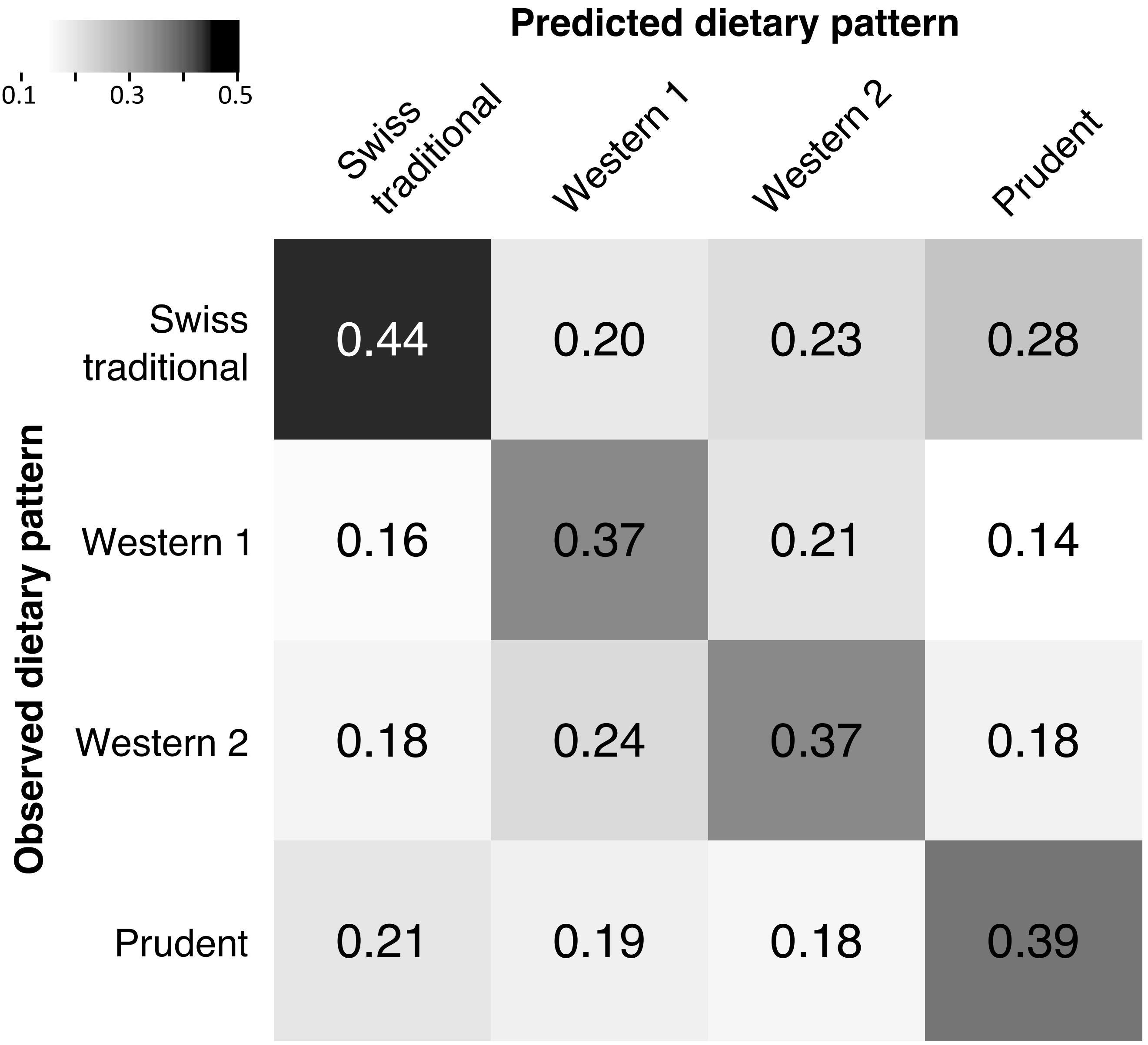

Supplementary figure 3: Confusion matrix of the multinomial logistic regression on the determinants of dietary patterns

Figures indicate the percentage of individuals following a dietary pattern among those predicted to follow a certain dietary pattern by the multinomial logisitic regression.
